# Prevalence and risk factors for hypertension and association with ethnicity in Nigeria: results from a national survey

**DOI:** 10.5830/CVJA-2013-058

**Published:** 2013-11

**Authors:** Gudlavalleti VS Murthy, Samantha Fox, Selvaraj Sivasubramaniam, Clare E Gilbert, Abdull M Mahdi, Abdullahi U Imam, Gabriel Entekume, Abiose Adenike, Olufunmilayo O Bankole, C Ezelum, Fatima Kyari, Mansur M Rabiu, Hannah Faal, Pak Sang Lee, Abubakar Tafida

**Affiliations:** Indian Institute of Public Health, Public Health Foundation of India, Hyderabad, Andhra Pradesh, India; International Centre for Eye Health, Department of Clinical Research, London School of Hygiene and Tropical Medicine, Keppel Street, London, UK; International Centre for Eye Health, Department of Clinical Research, London School of Hygiene and Tropical Medicine, Keppel Street, London, UK; International Centre for Eye Health, Department of Clinical Research, London School of Hygiene and Tropical Medicine, Keppel Street, London, UK; Abubakar Tafawa Balewa University Teaching Hospital, Bauchi, Nigeria; Ministry of Health, Minna, Niger State, Nigeria; Vision Health Services, Ikeja, Lagos State, Nigeria; Nigeria National Blindness and Visual Impairment study group: International Agency for Prevention of Blindness, Africa region, Ibadan, Nigeria; Ophthalmology Department, Lions Eye Centre, Isolo General Hospital, Lagos Sate, Nigeria; Ministry of Health Awka, Anambra State, Nigeria; National Eye Centre, Kaduna, Nigeria; National Eye Centre, Kaduna, Nigeria; Sight Savers West Africa Regional Office, Accra, Ghana; Institute of Ophthalmology, University College London, London, UK; Ministry of Health, Dutse, Jigawa State Nigeria

**Keywords:** hypertension, ethnicity, Nigeria, survey

## Abstract

**Background:**

Non-communicable diseases are now a global priority. We report on the prevalence of hypertension and its risk factors, including ethnicity, in a nationally representative sample of Nigerian adults recruited to a survey of visual impairment.

**Methods:**

A multi-stage, stratified, cluster random sample with probability proportional to size procedures was used to obtain a nationally representative sample of 13 591 subjects aged ≥ 40 years. Of these, 13 504 (99.4%) had a blood pressure measurement.

**Results:**

The prevalence of hypertension was 44.9% [95% confidence interval (CI): 43.5–46.3%]. Increasing age, gender, urban residence and body mass index were independent risk factors (*p* < 0.001). The Kanuri ethnic group had the highest prevalence of hypertension (77.5%, 95% CI: 71.0–84.0%).

**Conclusions:**

The high prevalence of hypertension in Nigeria is a cause for concern and suggests that it is inevitable that the impact of hypertension-related ill health is imminent, with the accompanying financial and societal costs to families and the state of Nigeria.

## Abstract

Hypertension is increasingly being recognised as an important public health problem in sub-Saharan Africa, with 26.9% of men and 28.4% of women in 2000 being estimated to have hypertension.^1^ Although lower than the prevalence in high-income countries (37.4% in men and 37.2% in women), in terms of numbers of people affected, the burden of hypertension in low- and middle-income countries is greater due to the large population.^1^

Hypertension has been recognised as a strong independent risk factor for heart disease and stroke and a predictor of premature death and disability from cardiovascular complications.^2^ It has been reported that 13.5% of deaths and 6% of disability-adjusted life years (DALYs) were attributed to hypertension globally, and for low- and middle income people, these figures were 12.9 and 5.6%, respectively over the period 1990 to 2001.^3^ Although infectious diseases remain the leading cause of mortality and morbidity in sub-Saharan Africa, the prevalence of cardiovascular disease and hypertension is rising rapidly.^4^

It has been emphasised that urbanisation is a key reason for the increasing rates of hypertension, as evidenced by the higher prevalence of hypertension in urban areas.^4^-^6^ Urban lifestyles, characterised by sedentary living, increased salt intake, obesity and stress contribute to these differences.^5^ With the urban population in sub-Saharan Africa projected to increase, a greater risk of hypertension is anticipated.

Studies on the association between ethnicity and hypertension in high-income countries have documented a higher prevalence of hypertension in black ethnic groups compared to white ethnic groups.^7^-^9^ Reasons for this association are complex, unclear and much debated, reflecting genetic and biochemical mechanisms, and environmental and socio-economic factors.^10^,^11^ There is limited evidence regarding differences in the prevalence of hypertension between ethnic groups within the broader classification of black ethnicity.^6^,^12^,^13^

Studies in Nigeria and sub-Saharan Africa have mainly involved specific geographical areas or have focused on sub-groups of the population.^5^,^14^ Surveys from Nigeria report prevalence estimates ranging from 20.2 to 36.6%, but all have involved participants with different age ranges.^15^-^18^ To plan services for hypertension in Nigeria, it is essential to have accurate prevalence estimates for the whole population and to identify populations at risk.

Nigeria, which is the most populous country in sub-Saharan Africa, is home to over 250 different ethnic groups. Nigeria is experiencing rapid urbanisation of the population, which is likely to increase the population at risk for hypertension.^19^ The present study is one of the largest population-based surveys in the region and is able to provide a nationally representative estimate of hypertension for Nigeria.

## Methods

As part of the Nigerian national blindness and visual impairment survey of adults aged 40 years and older, data were collected on blood pressure. A detailed description of the sampling, enumeration, visual acuity and ocular examination procedures has been published previously.^20^

Nigeria is divided into six administrative zones, which are called geo-political zones (GPZ), 36 states and the federal capital territory of Abuja. Each state is subdivided into local government authorities (LGA), which are the smallest administrative unit. There are 774 LGAs in the country.

A nationally representative sample was achieved through a multi-stage, stratified (by urban/rural location and GPZ), cluster random sampling with probability proportional to size procedures. A total of 310 clusters were identified, 226 were in rural areas and 84 in urban populations. The sample covered all 36 states. Fifty adults aged 40 years or older, who were normal residents (defined as being continually resident for at least the last three months) were randomly identified in each cluster.

These people were identified by the enumeration team who, having found the centre of the cluster, spun a bottle and approached the first household in the direction the bottle pointed. The team, using an established protocol, travelled from house to house to identify eligible adults, until the quota was achieved. If fewer than 50 were identified, the search continued into the next village. Five clusters were not included due to civil unrest or refusal to participate. Basic demographic data and informed consent were taken from each person who agreed to take part.

Respondents were invited to attend a clinical station which was set up in each cluster. Enumerated individuals not reporting to the clinical station were followed up three times and offered an examination at their house. If they still did not take part they were deemed non-respondents and were not replaced.

At the clinical station, individuals were interviewed to collect data on socio-demographic variables, including ethnic group, and history of disease and medication. All had anthropometric measures taken and their blood pressure was measured prior to ophthalmic examination. Each of the two survey teams had two qualified ophthalmologists and two ophthalmic nurses, who were recruited from each GPZ, so that they would know the main local languages.

Hypertension was measured by a qualified ophthalmic nurse trained in the procedure, using an Omron wrist instrument (UB322, Omron Healthcare Ltd, Milton Keynes, England), with the occluding cuff being placed around the volar surface of the wrist. The instrument was calibrated every morning. A total of three readings were taken by a trained nurse, at least five minutes apart after at least 10 minutes’ rest in a sitting position. The nurses’ performance was regularly monitored by the senior investigators. The mean of the three readings was calculated as the individual’s blood pressure.

Initial training was undertaken over two weeks and training sessions were repeated for each GPZ (two weeks each). A pilot study was conducted before the field work started in each GPZ. Inter-observer agreement studies were conducted periodically throughout the study for the ophthalmic nurses and the ophthalmologists. Data were collected over a 30-month period from January 2005 to July 2007.

Height and weight were measured using a Tanita measurement scale (Model 1536, Tanita Corporation, Tokyo, Japan). The weight measure was calibrated every morning and checked for error using a standard weight.

The World Health Organisation’s (WHO) classification of hypertension was used. Hypertension is defined as diastolic blood pressure (DBP) of 90 mmHg or greater, or a systolic blood pressure (SBP) of 140 mmHg or greater. The WHO grading system of hypertension to profile risk was also used. This defines grade 1 hypertension as SBP 140–159 mmHg or DBP 90–99 mmHg; grade 2 as SBP 160–179 mmHg or DBP 100–109 mmHg; and grade 3 as SBP ≥ 180 or DBP ≥110 mm Hg. These grades are used to assess the risk in individuals for a cardiac event in the context of other additional risk factors.^21^

Body mass index (BMI) was calculated from weight (kg) divided by height (m) squared. World Health Organisation categories of BMI were used in the analysis.^22^

Socio-economic status (SES) was calculated by assigning one of eight occupational categories, ranging from 0 (not in gainful employment) to 7 (professional), and a grade for the highest level of school attended, from 0 (no schooling) to 4 (university) for each person. The sum of the scores was calculated, with a higher score signifying a more affluent socio-economic status. The ranked SES scores were then divided into tertiles.

Data on ethnic group were categorised such that groups with more than 100 participants were analysed separately, with all other ethnic groups combined into an ‘other’ group. Ethnic groups were classified based on the father’s ethnic status.

Ethical approval for the study was provided by the London School of Hygiene and Tropical Medicine and the Federal Government of Nigeria. The study adhered to the tenets of the Declaration of Helsinki. Written informed consent was obtained from participants after an explanation of the nature of the study.

## Statistical analysis

A customised database was created in Microsoft Access and two trained data-entry clerks entered the data. Data entered by one operator were checked by the second operator and corrections were made when necessary. Quality assurance procedures included a random verification of filled forms completed in the field and at the project office. The data were cleaned and analysed using a statistical package (Stata 11; StataCorp, College Station, TX).

Prevalence estimates together with 95% confidence intervals (CI) for hypertension are presented. Multiple logistic regression analysis was performed to identify risk factors for hypertension and to estimate adjusted odds ratios (OR). All the analyses accounted for clustering (design effect) due to the cluster sampling design adopted for the study. Missing values were excluded from all the analyses; *p*-values < 0.05 were considered statistically significant.

## Results

A total of 13 591 people took part in the study, 13 504 (99.4%) of whom had a valid blood pressure measurement. The mean age of participants was 55.9 years (± 12.4) (men: 56.8; women: 55.2 years). The mean age of respondents in the south-east geo-political zone was the highest (58.6 ± 12.8 years) and it was the lowest in the north-east zone (53.8 ± 11.6 years). The mean age in the north-west was 54.2 years (± 11.8), in north-central it was 55.8 years (± 12.7), in south-south it was 56 years (± 12.2) and in south-west, 57.8 years (± 11.8). Over 60% of those surveyed were under the age of 60 years.

There were more females in the sample (54.1%) than males, and rural respondents constituted 77.6% of the sample (Table 1). Over 50% of the study population belonged to the four most populous ethnic groups in Nigeria: Hausa, Igbo, Yoruba and Fulani.

**Table 1:** Demographic Characteristics And Mean Blood Pressure Of The Study Population

*Parameters surveyed*	*Frequency (n)*	*Percent (%)*	*Mean systolic BP (mmHg) (SD)*	*Mean diastolic BP (mm Hg) (SD)*
	13 504		138.2 (25.9)	83.9 (15.3)
Age groups (years)				
40–49	4 858	36.0	130.2 (21.8)	81.9 (14.2)
50–59	3 554	26.3	39.3 (26.0)	85.1 (15.4)
60–69	2 758	20.4	144.0 (27.0)	85.2 (15.8)
70–79	1 643	12.2	146 (27.6)	84.4 (16.3)
≥ 80	691	5.1	146.3 (28.6)	84.4 (16.6)
Gender				
Male	6 203	45.9	136.8 (24.6)	83.4 (15.0)
Female	7 301	54.1	139.3 (27.0)	84.2 (15.6)
Residence				
Rural	10 478	77.6	137.2 (25.5)	83.1 (15.0)
Urban	3 026	22.4	141.6 (27.1)	86.6 (16.1)
Literacy				
Literate	5 891	43.6	136.4 (25.1)	83.7 (15.3)
Illiterate	7 613	56.4	139.5 (26.5)	84.0 (15.3)
Geo-political zone				
North-east	1 707	12.6	144.4 (27.7)	90.4 (16.5)
South-east	1 657	12.3	138.4 (28.0)	79.0 (14.8)
South-south	1 845	13.7	133.1 (25.9)	77.9 (14.8)
North-west	3 577	26.5	140.3 (24.3)	87.5 (13.7)
South-west	2 708	20.1	137.1 (26.4)	81.9 (15.0)
North-central	2 010	14.9	135.0 (23.4)	83.9 (14.5)
Socio-economic status				
Most affluent	4 383	32.5	135.2 (24.5)	82.5 (14.7)
Moderately affluent	4 676	34.6	137.2 (25.4)	82.9 (14.9)
Least affluent	4 445	32.9	142.1 (27.4)	86.3 (16.1)
Body mass index				
Underweight	1 495	11.1	134.3 (25.9)	81.2 (15.8)
Normal	8 156	60.4	136.1 (25.1)	82.7 (14.9)
Overweight	2 588	19.2	142.3 (26.1)	86.4 (15.2)
Obese	1 113	8.2	147.9 (27.5)	89.8 (15.4)
Missing data	152	1.1		

The overall prevalence of hypertension was 44.9% (95% CI: 43.5–46.3%). The prevalence of grade 3 hypertension (the most severe grade) was 9.9% (95% CI: 9.2–10.6%) (Table 2). Among the 6 064 respondents who were diagnosed as having any hypertension, only 14.1% (854) knew previously that they were hypertensive. Among the 2 975 respondents who were observed to have grade 2 or 3 hypertension, 19.8% (588) were aware of their hypertension status.

**Table 2:** Prevalence Of Hypertension And Association With Socio-Demographic Factors

	*Any hypertension SBP ≥ 140, DBP ≥ 90 mmHg*			*Grade 2 and 3 hypertension SBP ≥ 160, DBP ≥ 100 mmHg*			*Grade 3 hypertension SBP ≥ 180, DBP ≥ 110 mmHg*		
*Parameters*	*OR*	*95% CI*	p*-value*	*OR*	*95% CI*	p*-value*	*OR*	*95% CI*	*p-value*
All ages (13 504)	44.7	43.5–46.3	22.0	20.9–23.2	9.9	9.2–10.6
Age group (years)						
40–49 (4 858)	32.1	30.2–33.9	12.8	11.6–14.1	5.4	4.6–6.2
50–59 (3 554)	47.1	44.9–49.2	23.4	21.5–25.2	10.3	9.1-11.4
60–69 (2 758)	54.2	52.1–56.3	28.4	26.6–30.3	12.8	11.5–14.1
70–79 (1 643)	57.1	54.2–59.9	31.2	28.7–33.6	14.9	13.0–16.8
≥ 80 (691)	58.2	54.4–61.9	32.9	29.4–36.3	15.9	13.1–18.7
	*p* < 0.0001		*p* < 0.0001		*p* < 0.0001	
Gender						
Male (6 203)	42.6	40.9–44.4	19.2	17.8–20.5	8.5	7.7–9.3
Female (7 301)	46.8	45.3–48.4	24.5	23.1–25.9	11.1	10.1–12.0
	*p* < 0.0001		*p* < 0.0001		*p* < 0.0001	
Residence						
Rural	43.0	41.3–44.6	20.5	19.2–21.8	9.1	8.3–9.8
Urban	51.6	48.4–54.9	27.3	24.5–30.2	12.8	10.9–14.8
	*p* < 0.0001		*p* < 0.0001		*p* < 0.0001	
Geopolitical zone						
North-east (1 707)	60.5	55.1–65.8	33.5	28.7–38.2	16.5	13.1–20.0
South-east (1 657)	41.0	38.0–44.0	20.7	18.4–23.0	9.9	8.3–11.5
South-south (1 845)	34.2	31.2–37.2	17.0	14.6–19.4	7.0	5.4–8.7
North-west (3 577)	51.5	48.7–54.2	24.2	21.6–26.8	10.1	8.5–11.6
South-west (2 708)	40.1	37.1–43.2	19.7	17.4–22.0	9.2	7.9–10.5
North-central (2 010)	39.5	35.7–43.3	17.4	14.8–19.9	7.6	6.2–8.9
	*p* < 0.0001		*p* < 0.0001		*p* < 0.0001	
Socio-economic status						
Affluent (4 383)	39.2	37.4–41.0	18.0	16.7–19.3	7.7	6.8–8.6
Moderately affluent (4 676)	43.5	41.4–45.5	20.0	18.5–21.6	8.8	7.8–9.8
Least affluent (4 445)	52.1	49.9–54.2	28.1	26.1–30.1	13.3	11.8–14.7
	*p* < 0.0001		*p* < 0.0001		*p* < 0.0001	
Body mass index (13 352)						
Underweight (1 495)	39.6	36.6–42.6	18.7	16.5–21.0	8.5	6.9–10.1
Normal (8 156)	41.7	40.1–43.2	19.5	18.3–20.7	8.5	7.7–9.3
Overweight (2 588)	50.9	48.7–53.2	26.0	24.0–28.0	11.9	10.5–13.3
Obese (1 113)	59.8	56.3–63.2	34.2	31.1–37.4	16.2	13.9–18.4
	*p* < 0.0001		*p* < 0.0001		*p* < 0.0001	

The prevalence of hypertension increased with age, ranging from 32.1% in 40–49 year olds to 58.2% in those aged ≥ 80 years (Table 3). The prevalence was higher in females (46.8%) and in urban participants (51.6%). The prevalence was also higher among illiterate respondents (47.2%). There was a wide variation in prevalence according to GPZ, the lowest being in the south-south GPZ (34.2%), and highest in the north-east GPZ (60.5%).

**Table 3:** Association Between Hypertension And Specific Risk Factors (Univariate Analysis)

	*Any hypertension SBP ≥ 140, DBP ≥ 90 mmHg*			*Grade 2 and 3 hypertension SBP ≥ 160, DBP ≥ 100 mmHg*			*Grade 3 hypertension SBP ≥ 180, DBP ≥ 110 mmHg*		
*Parameters*	*OR*	*95% CI*	p*-value*	*OR*	*95% CI*	p*-value*	*OR*	*95% CI*	*p-value*
Age group (years)									
40–49	1.00			1.00			1.00		
50–59	1.88	1.71–2.07	< 0.001	2.08	1.84–2.34	< 0.001	1.99	1.67–2.38	< 0.001
60–69	2.51	2.27–2.77	< 0.001	2.70	2.39–3.06	< 0.001	2.55	2.13–3.06	< 0.001
70–79	2.82	2.47–3.22	< 0.001	3.08	2.65–3.59	< 0.001	3.05	2.50–3.72	< 0.001
≥ 80	2.95	2.49–3.49	< 0.001	3.33	2.79–3.98	< 0.001	3.29	2.60–4.18	< 0.001
Gender									
Male	1.00			1.00			1.00		
Female	1.19	1.10–1.27	< 0.001	1.37	1.26–1.49	< 0.001	1.33	1.18–1.50	< 0.001
Residence									
Rural	1.00			1.00			1.00		
Urban	1.42	1.21–1.66	< 0.001	1.46	1.23–1.73	< 0.001	1.48	1.20–1.82	< 0.001
Literacy									
Literate	1.00			1.00			1.00		
Illiterate	1.23	1.13–1.35	< 0.001	1.24	1.12–1.38	< 0.001	1.23	1.09–1.40	< 0.001
Geo-political zones									
North-east	2.94	2.27–3.81	< 0.001	2.46	1.87–3.24	< 0.001	2.61	1.84–3.71	< 0.001
South-east	1.34	1.11–1.60	0.002	1.28	1.03–1.59	0.029	1.45	1.07–1.96	0.017
South-south	1.00			1.00			1.00		
North-west	2.04	1.72–2.42	< 0.001	1.56	1.25–1.95	< 0.001	1.48	1.10–2.0	0.011
South-west	1.29	1.07–1.55	0.006	1.20	0.96–1.51	0.108	1.33	0.99–1.78	0.056
North-central	1.26	1.02–1.54	0.03	1.03	0.80–1.32	0.824	1.08	0.79–1.48	0.635
Socio-economic status									
Affluent	1.00			1.00			1.00		
Moderately affluent	1.19	1.08–1.32	< 0.001	1.14	1.02–1.28	0.025	1.15	0.98–1.35	0.084
Least affluent	1.69	1.52–1.87	< 0.001	1.78	1.58–2.01	< 0.001	1.83	1.55–2.15	< 0.001
Body mass index (13 352)									
Underweight	0.92	0.81–1.04	0.163	0.95	0.83-1.10	0.489	1.00	0.82-1.22	0.998
Normal	1.00			1.00			1.00		
Overweight	1.45	1.32–1.6	< 0.001	1.45	1.30–1.62	< 0.001	1.45	1.27–1.66	< 0.001
Obese	2.08	1.78–2.43	< 0.001	2.15	1.85–2.50	< 0.001	2.08	1.73–2.50	< 0.001

The prevalence of hypertension increased from the most affluent (39.2%) to the least affluent (52.1%). The prevalence of hypertension also increased with BMI category; 39.6% of those who were underweight had hypertension compared with 59.8% in those who were obese. The same trends were noted with grades 2 and 3, and grade 3 hypertension.

With univariate analysis, age, female gender, living in an urban area and being illiterate were strongly associated with all grades of hypertension (*p* < 0.001) (Table 3). Using the south-south GPZ as the baseline, associations with GPZ were more variable. Socio-economic status was inversely associated with hypertension (*p* < 0.001); the odds ratio of hypertension were inversely associated with affluence. Compared with those with a normal BMI, being overweight, obese or underweight were all associated with hypertension.

Multivariate analysis was undertaken using the same set of variables, excluding literacy due to its correlation with the measure of SES (Table 4). In this analysis, older age, female gender, urban area of residence and high and low BMI were independently associated with hypertension at the *p* < 0.001 level, with the same trends within categories as in the univariate analysis. Findings with regard to GPZ showed that in relation to the south-south, participants living in all other GPZs had a significantly higher odds ratio of hypertension, apart from those in the south-west.

**Table 4:** Association Between Hypertension And Specific Risk Factors In Multivariate Analysis*

	*Any hypertension SBP ≥ 140, DBP ≥ 90 mmHg*			*Grade 2 and 3 hypertension SBP ≥ 160, DBP ≥ 100 mmHg*			*Grade 3 hypertension SBP ≥ 180, DBP ≥ 110 mmHg*		
*Parameters*	*OR*	*95% CI*	p*-value*	*OR*	*95% CI*	p*-value*	*OR*	*95% CI*	*p-value*
Age group (years)									
40–49	1.00			1.00		0.001	1.00		< 0.001
50–59	2.07	1.88–2.29	< 0.001	2.24	1.99–2.54	< 0.001	2.08	1.73–2.49	< 0.001
60–69	3.24	2.93–3.58	< 0.001	3.39	2.99–3.84	< 0.001	2.99	2.48–3.61	< 0.001
70–79	3.91	3.41–4.49	< 0.001	4.10	3.51–4.80	< 0.001	3.80	3.06–4.72	< 0.001
≥ 80	4.24	3.51–5.13	< 0.001	4.63	3.79–5.65		4.20	3.24–5.44	
Gender									
Male	1.00			1.00			1.00		
Female	1.26	1.16–1.37	< 0.001	1.44	1.31–1.58	< 0.001	1.36	1.18–1.56	< 0.001
Residence									
Rural	1.00			1.00			1.00		
Urban	1.31	1.14–1.49	< 0.001	1.32	1.12–1.54	< 0.001	1.33	1.10–1.6	< 0.003
Geo-political zones									
North-east	3.81	2.94–4.92	< 0.001	3.14	2.40–4.1	< 0.001	3.16	.24–4.45	< 0.001
South-east	1.27	1.06–1.53	0.012	1.21	0.97–1.5	0.094	1.35	1.00–1.82	0.053
South-south	1.00			1.00			1.00		
North-west	2.53	2.14–2.98	< 0.001	1.89	1.52–2.36	0.001	1.74	1.29–2.34	< 0.001
South-west	1.16	0.98–1.38	0.090	1.07	0.86–1.33	0.536	1.20	0.90–1.59	0.208
North-central	1.29	1.05–1.58	0.016	1.04	0.81–1.03	0.766	1.07	0.79–1.46	0.644
Socio-economic status									
Affluent	1.00			1.00			1.00		
Moderately affluent	0.97	0.88–1.06	0.473	0.91	0.81–1.03	0.130	0.94	0.80–1.11	0.467
Least affluent	1.09	0.97–1.22	0.128	1.14	1.00–1.29	0.056	1.17	0.99–1.39	0.063
Body mass index (13 352)									
Underweight	0.68	0.60–0.77	< 0.001	0.69	0.60–0.8	< 0.001	0.74	0.61–0.9	0.003
Normal	1.00			1.00			1.00		
Overweight	1.69	1.53–1.86	< 0.001	1.63	1.46–1.82	< 0.001	1.59	1.39–1.82	< 0.001
Obese	2.61	2.23–3.05	< 0.001	2.53	2.16–2.97	< 0.001	2.35	1.92–2.87	< 0.001

*Literacy was excluded from the multivariate model as it was associated with socioeconomic status.

Ethnicity data were missing for 58 participants and they were excluded from this analysis. The prevalence of hypertension varied by ethnic group, with the highest prevalence being in the Kanuri group (77.5%) and lowest in the Gbagyi group (25.9%) (Table 5). Adjusting for age, gender, place of residence and socio-economic status, the Kanuri, Nupe, Tiv, Fulani, Anang, Hausa and Edo ethnic groups had statistically significantly greater odds ratios of hypertension than the ‘other’ group. The Yoruba group had statistically significantly lower odds ratios of hypertension than the ‘other’ group.

**Table 5:** All Grades Of Hypertension Among Those Aged ≥ 40 Years In Nigeria, By Ethnic Group

*Ethnic group*	*n*	*Mean age ± SD (years)*	*Prevalence (%)*	*95% CI*	*Adjusted OR*	*95% CI*	p*-value*
Other group	2 024	54.7 ± 12.1	41.5	37.5–45.5	1		
Kanuri	333	54.4 ± 11.7	77.5	71.0–84.0	3.51	2.33–5.29	< 0.001
Nupe	208	53.9 ± 10.8	50.5	43.9–57.1	1.85	1.14–3.0	0.01
Tiv	342	55.9 ± 13.7	44.4	37.7–51.2	1.65	1.14–2.38	0.01
Igebe	143	54.8 ± 12.1	37.8	24.8–50.7	1.64	0.97–2.77	0.06
Fulani	832	54.1 ± 11.3	54.6	49.4–59.7	1.47	1.11–1.95	0.01
Ibibio	210	52.6 ± 11.5	38.6	30.1–47.0	1.45	0.99–2.13	0.06
Anang	132	54.2 ± 11.1	34.1	27.5–40.6	1.45	1.06–1.98	0.02
Hausa	3 361	54.4 ± 11.9	52.4	49.5–55.2	1.42	1.14–1.76	< 0.001
Igala	183	55.6 ± 12.9	41.5	33.5–49.6	1.38	0.83–2.30	0.22
Edo	218	56.6 ± 12.4	39.0	35.8–42.2	1.32	1.01–1.72	0.05
Igbirra	163	56.7 ± 13.9	42.3	36.4–48.2	1.22	0.83–1.8	0.30
Ijaw	251	58.3 ± 12.9	39.4	28.8–50.1	1.07	0.69–1.67	0.75
Igbo	2 017	57.5 ± 12.7	40.4	37.6–43.1	1.04	0.77–1.40	0.78
Ekoi	103	57.3 ± 11.0	31.1	27.1–35.0	1.04	0.79–1.36	0.80
Urhobo	266	57.9 ± 13.4	36.1	27.1–45.0	0.94	0.58–1.53	0.81
Yoruba	2 525	58.5 ± 12.4	39.3	36.4–42.1	0.79	0.63–0.99	0.04
Gbagyi	135	54.8 ± 14.3	25.9	18.3–33.6	0.73	0.42–1.26	0.26

*Adjusted for age, gender, place of residence and socio-economic status.

## Discussion

The prevalence of hypertension reported in this survey was higher than in other smaller studies carried out in Nigeria, where the prevalence ranged from 20.2 to 36.6%.^15^,^23^-^26^ However the age range of respondents as well as definitions and methods used differed between studies. A systematic review of published data from 43 studies in Nigeria found that the prevalence of hypertension ranged between 8 and 46.4% depending on the study population and definitions used.^16^

A number of recent large surveys in sub-Saharan Africa reported the prevalence of hypertension to range from 19 to 50.1%,^6^,^12^,^27^-^34^ and a recent systematic review of surveys from the region confirms the wide variation in prevalence estimates.^5^ Prevalence of hypertension reported from different studies in Africa shows that Nigeria has a high prevalence rate for hypertension (Table 6). Some of the variation can be explained by methodological differences (e.g. some have focused on only rural populations) but variation in the age groups studied is likely to be a major factor.

**Table 6:** Estimates Of Hypertension From Population-Based Studies In Sub-Saharan Africa

*Author (year)*	*Place of study*	*Sample size*	*Age range (years)*	*Prevalence (definition 1) (%)*	*Prevalence (definition 2) (%)*
Present study	Nigeria, nationwide	13 504	40+	45.9*	24.3*
Isezuo *et al.*^18^ (2011)	Nigeria (rural and urban)	782	15–65	24.5	Nigeria (rural)
Oladapo *et al.*^19^ (2010)	Nigeria (rural)	2 000	18–64	20.8	
Adedoyin *et al.*^20^ (2008)	Nigeria (semi-urban)	2 097	20+	36.6*	
Omuemu *et al.*^21^ (2007)	Nigeria (rural)	590	15+	20.2*	
Olatunbosun *et al.*^29^ (2000)	Nigeria, (urban)	998	16–70		10.4
Cooper *et al.*^13^ (1997)	Nigeria (rural and urban)	1 171	25+		14.5*
Andy *et al.*^15^ (2012)	Nigeria (rural)	3 869	15+	23.6	
Ogah *et al.*^18^ (2013)	Nigeria (rural)	2 999	18+	31.8	
Ekpenyong *et al.*^38^ (2010)	Nigeria (rural)	2 780	18-60	25	
Maher *et al.*^25^ (2011)	Uganda (rural)	6 678	All	22.5*	
De Ramirez *et al.*^12^ (2010)	Malawi, Rwanda, Tanzania (rural)	1 485	18+	22	
Mathenge *et al.*^6^ (2010)	Kenya (rural and urban)	4 396	50+	50.1	
Damasceno *et al.*^23^ (2009)	Mozambique (rural and urban)	3 323	25–64	33.1	
Tesfaye *et al.*^26^ (2009)	Ethiopia (urban)	3 173	25–64	31	
Addo *et al.*^27^ (2008)	Ghana (urban)	1 015	25+	30.3	
Kengne *et al.*^28^ (2007)	Cameroon (urban)	2 559	15+	20.8	
Steyn *et al.*^24^ (2001)	South Africa, nationwide	13 802	15+	23.9	
Van der Sande^22^ (2000)	Gambia	5 369	15+	19	
Dewhurst *et al.*^39^ (2011)	Tanzania (rural Hai)	2 223	70+	69.9	
Houinato *et al.*^40^ (2008)	Benin		6 853	25–64	27.9
Hendriks *et al.*^41^ (2009–11)	Nigeria, Kenya (rural), Tanzania, Namibia (urban)	7 568		18+	Nigeria: 19.3
				Kenya: 21.4	
				Tanzania: 23.7	
				Namibia: 38	

*Medication details not available Definition 1: Diastolic BP 140 mmHg ≥ or SBP ≥ 90 mmHg Definition 2: Diastolic BP 160 mmHg ≥ or SBP ≥ 95 mmHg

A limitation of our study was the non-availability of data on the use of anti-hypertensive medication in the present study. However, studies in Nigeria and other sub-Saharan African countries report that use of hypertensive medication was very low,^5^,^13^,^23^,^27^,^32^ and so this is unlikely to have significantly impacted on our prevalence estimate or the findings of association in the present study. If there was any bias, our estimate would be an under-estimate.

We observed that women had a higher prevalence of hypertension than men in Nigeria. This corroborates the findings of many,^4^,^5^,^24^,^27^,^35^ but not all studies in Africa.^28^,^32^,^34^

A unique feature of the present study is that it provides estimates of the prevalence of hypertension among indigenously resident ethnic groups at a national level. Only one other study examined the association of ethnicity with hypertension in Nigeria but it was limited to one region of the country.^15^

Some evidence of ethnic variation has been reported in Kenya where statistically significant differences between ethnic groups were reported after adjusting for socio-demographic and other cardiovascular risk factors,^6^ but a study from Nigeria and Cameroon did not find any association of hypertension with ethnicity.^13^ A review of existing studies to estimate the prevalence of hypertension in 11 sub-Saharan African countries found clear differences by country, which the authors suggested may be partly explained by ethnicity and other socio-demographic factors.^14^ The very high prevalence of hypertension among some ethnic groups such as the Kanuri in Nigeria needs further investigation as this ethnic group is concentrated in one localised region of the country.

Our results show that the prevalence of hypertension in Nigeria is similar to that in high-income countries and is therefore a public health challenge. A review of published studies from Nigeria observed that the pooled prevalence of hypertension increased from 8.6% over the period 1970–1979 to 22.5% over the period 2000–2011.^16^ A national survey on non-communicable diseases in Nigeria documented that the prevalence of hypertension using a cut-off value of 160/95 mmHg was 11.2% (age-adjusted prevalence was 9.3%).^36^

The observed trend shows that Nigeria is at high risk of a significant increase in rates of hypertension in the near future. Recent evidence documents that hypertension is the commonest condition seen at medical centres in Nigeria.^37^ This has implications on mortality of people during the productive years of their lives. A study by Ekpenyong *et al.* estimated that five million Nigerians would die of non-communicable diseases (NCDs) in Nigeria alone.^38^ A significant proportion of these deaths may be contributed to by hypertension.

## Conclusion

The increase in NCDs in sub-Saharan Africa will mean that additional resources will be required for the detection and control of NCDs, which would compete with the resources being allocated for the control of communicable diseases, such as the neglected tropical diseases, malaria and HIV. Strategies and interventions will also be required to improve adherence to life-long medication. It is inevitable that hypertension and its consequences will lead to financial and societal costs to families and the states in sub-Saharan Africa and governments will need to respond to this emerging challenge.
